# Body shape and robustness response to water flow during development of brown trout Salmo trutta parr

**DOI:** 10.1111/jfb.13772

**Published:** 2018-09-21

**Authors:** Miriam Fenkes, Holly A. Shiels, Robert L. Nudds

**Affiliations:** ^1^ University of Manchester, Faculty of Biology Medicine and Health, School of Biological Sciences Manchester UK

**Keywords:** exercise, geometric morphometrics, morphology, re‐stocking, Salmo trutta

## Abstract

Domesticated brown trout Salmo trutta parr were subjected to increased, variable flow under controlled experimental conditions. Using geometric morphometric analyses, $\bar{K}\;$(a mass–length index) and caudal fin area–body length ratio, this study assessed morphological responses in lateral body depth, growth and robustness and propulsive potential, respectively, of parr over the course of 32 weeks. Geometric morphometric analyses did not reveal an effect of exercise on either lateral body depth or caudal fin area. However, improved overall robustness and growth trajectories in exercised parr showed a positive adaptive response to the enriched habitat. Exercise and habitat heterogeneity thus have the potential to improve survivability of domesticated salmonids in the wild.

## 1 INTRODUCTION

Global warming driven by human‐induced, accelerated climate change is predicted to threaten the persistence of unprecedented numbers of terrestrial and aquatic species (IPCC, 2013, 2014). Freshwater species face especially acute challenges due to the additional stressors of anthropogenic habitat modification, pollution and intense commercial exploitation (Brander, 2007; Budy *et al*., 2002; IPCC, 2014). As a result, many species are in decline, including ecologically and socio‐economically important fishes like salmonids (Almodóvar *et al*., 2012 *;* Bryant, 2009 *;* Crozier *et al*., 2008 *;* Farrell *et al*., 2008 *;* Hinch *et al*., 2012 *;* ICES, 2017a, 2017b *;* IPCC, 2014).

Increasingly, supplementation of wild fish populations through artificially propagated and captive‐reared individuals is used as a restorative tool to increase the number of reproducing adults, as well as for the purpose of harvest augmentation (Berejikian *et al*., 1997 *;* Christie *et al*., 2012 *;* ICES, 2015, 2017a, 2017b *;* Molony *et al*., 2003), provided that prior habitat assessments confirm sufficient carrying capacity of the target ecosystem (Molony *et al*., 2003). Such stock enhancement has been implemented for over a century (Molony *et al*., 2003), but the practice remains controversial. Known adverse effects include genetic introgression by behaviourally and genetically inferior domesticated individuals (Bessey *et al*., 2004; Clifford *et al*., 1998; Fitzpatrick *et al*., 2011; Lehnert *et al*., 2013; McGinnity *et al*., 2003), as well as competition for resources with wild stocks within continually decreasing suitable habitat (Fleming *et al*., 2002; Hard *et al*., 2000). In addition, many stock‐enhancement projects fail due to poor survival of the released fish (Molony *et al*., 2003). This is often attributable to significant behavioural deficits of hatchery‐reared fish such as reduced predator recognition and evasion and food acquisition capabilities (Olla *et al*., 1998). Besides behaviour, domestication and captive rearing also have demonstrable effects on salmonid growth and morphology. Growth and food conversion efficiency are traits directly targeted by artificial selection in domesticated salmonids, resulting in growth‐rate increases of up to 15% in some species (Fleming *et al*., 2002). Brown trout *Salmo trutta* L. 1758 reared in hatcheries have shorter heads and are less streamlined compared with individuals from natural rearing habitat (Vehanen & Huusko, 2011), while hatchery reared Atlantic salmon *Salmo salar* L. 1758 parr have smaller heads, smaller rayed fins and narrower caudal peduncles than their wild counterparts (Fleming *et al*., 1994; but see Poole *et al*., 2003). Similar differences have been observed in wild and hatchery‐reared coho salmon *Oncorhynchus kisutch* (Walbaum 1972) and these changes in morphology may affect survival and growth in the wild mainly through altered streamlining (Fleming *et al*., 1994; von Cramon‐Taubadel *et al*., 2005).

One frequently evoked driver for this morphological variation is the marked difference in environmental stimuli between captive and wild rearing habitats, especially the lack of water flow in hatcheries. In the wild, most salmonid alevin are immediately exposed to lotic conditions upon emergence from the gravel of their natal tributaries and adjoining river systems (Hinch *et al*., 2005). The complex nature of their natural lacustrine or riverine habitats, especially with regard to thermal and water velocity fluctuations, has led to a high degree of adaptive phenotypic plasticity in salmonid species (Garcia de Leaniz *et al*., 2007). For example, Mclaughlin and Grant (1994) observed that newly‐emerged brook charr *Salvelinus fontinalis* (Mitchill 1814) found in fast‐flowing areas of rivers have longer bodies with large caudal fins, making them more fusiform, compared with individuals found in slow flowing areas. These individuals also differ in locomotory aspects of their foraging strategies, suggesting that the observed phenotypic plasticity is adaptive (Mclaughlin & Grant, 1994). The lack of habitat heterogeneity and divergent selective pressures in captivity, however, reduces genetic heterozygosity and allelic richness in domesticated fish (Blanchet *et al*., 2008; Clifford *et al*., 1998), reducing their potential for adaptation to changing environments. Captive rearing therefore often fails to prepare hatchery fish for the challenges within their natural habitats, decreasing their chances for survival upon (re‐) introduction (Anttila *et al*., 2011; Farrell *et al*., 1997).

Forced prolonged swimming at sustainable speeds (exercise training) has been implemented to improve various fitness related aspects of hatchery‐reared fish (Davison, 1997; Palstra & Planas, 2011). Besides improving their overall swimming performance (Anttila *et al*., 2008, 2011), welfare and condition (Palstra & Planas, 2011), exercise training has been shown to affect the growth and morphology of hatchery‐reared salmonids, albeit yielding contradictory results. For example, exposure to experimentally increased water flow caused young‐of‐the‐year *S. trutta* to become slightly more streamlined compared with individuals reared in a slow‐flowing environment, whereas *S. salar* grew more robust in shape when exposed to high flow (Pakkasmaa & Piironen, 2000). These results were observed after a short exposure to increased flow, demonstrating the high potential for developmental plasticity in these species (Pakkasmaa & Piironen, 2000). However, although hatched and reared in captivity, the individuals used by Pakkasmaa and Piironen (2000) were first generation offspring of wild adults. In light of their reduced genetic diversity (Blanchet *et al*., 2008), the question remains whether domesticated salmonids are capable of a similar phenotypic response to exercise training.

Here, the phenotypic response of domesticated *S. trutta* parr to increased and variable water flow under controlled experimental conditions was assessed over the course of 32 weeks. Three approaches measuring different aspects of fish body shape were used to determine whether water flow induces any aspect of morphological (and potentially physiological) change. One approach was a geometric morphometric analysis of the shape of the lateral aspect of the *S. trutta* parr. In sub‐carrangiform swimmers like salmonids, lateral body shape represents the size of the main propulsive surfaces and is therefore an indicator of propulsive efficiency and manoeuvrability (Webb, 1984). The second index focussed on the mass–length ratio of fish and was used to assess the overall robustness of the parr. Increased robustness (i.e., increased girth for a given length) is assumed to approximate improved physical condition and health (Froese, 2006). The final approach related the area of the caudal fin (the main propulsor) to body size. It was hypothesized that increased water flow would increase body depth, especially depth of the caudal peduncle region and relative caudal‐fin size of *S. trutta* parr. We also hypothesized that water flow would induce a change in overall fish robustness, but due to the aforementioned ambiguity in results from previous studies the directionality of this effect could not confidently be predicted.

## 2 MATERIALS AND METHODS

### 2.1 Experimental fish and rearing conditions

All‐female, diploid *S. trutta* at eyed egg stage were obtained from Dunsop Bridge Trout Farm Ltd (http://www.dunsop-bridge.cylex-uk.co.uk). Upon arrival, the eggs were transferred into egg tumblers (ZET‐55/ZET‐65 Ziss Tumbler; Ziss Aqua; http://www.zissaqua.eu) suspended in one constantly oxygenated 40 L rectangular fish tank maintained at an average incubation temperature of 6.4 ± 0.3°C. Dead eggs and alevin were removed daily to avoid fungal and bacterial contamination. Time between first and completed hatching was 12 days; the common hatching date was set midway between first and complete hatching (January 21, 2015). Upon completed hatching, the alevin were transferred into hatching baskets and the tank temperature gradually increased to 10.4 ± 0.5°C until the animals reached swim‐up stage. Increased flow and control treatments (still water) were initiated at age 97 days from hatching, when parr were independently swimming and feeding.

### 2.2 Training regime

For the purpose of sustained exercise training, the fish were divided into two cohorts, each transferred into one of two constantly oxygenated and filtered 90 I circular fish tanks. Each tank was fitted with a vertical tube element in its centre to create a circular swimming channel. One cohort (Exercise) was housed in a tank fitted with 800–1,600 l h^−1^ adjustable circulation pumps (NEWA Wave 1.6 adj.; NEWA Tecno Industria Srl; http://www.newa.it) at the bottom and near the surface of the water column, respectively, creating a current along the swimming channel. In addition, the external filter outlet in this tank was fitted with a jet nozzle directed into the flow. The second cohort (Control) was housed in a tank without circulation pumps and fitted with a perforated filter outlet pipe diffusing the water to minimize flow. Water temperature was recorded daily, with an experiment duration mean of 11.1 ± 0.7°C (Exercise tank) and 11.7 ± 0.9°C (Control tank), respectively, controlled by submersible cooling plates chilled by a central water bath. Water quality variables (pH, NH_3_, NO_2_^−^, NO_3_^−^) were monitored and remained within tolerance levels over the experimental period. Water flow was determined to the nearest 0.01 m s^−1^ in both tanks at the start of the experiment using a submersible flow probe (HFA, Höntzsch Instruments GmbH; http://www.hoentzsch.com) at 36 positions, covering three horizontal by three vertical depths at four equidistant positions along the circular water channel (Supporting Information S1 Flow speed measurements and Figure S1 in File S1). A total of eight repeated measurements, spread over the initial 12 weeks of treatment, were used to determine the overall difference in current speed between the tanks. From a linear mixed effects model (R 3.3.1 GUI 1.68 Mavericks build (http://www.r-project.org): lme4 package 1.1–12 (Bates *et al*., 2015) and car package 2.1–3 (Fox & Weisberg, 2011)), controlling for repeated measurements at each of the 36 positions, adjusted means ± s.e. (lsmeans package 2.23–5 (Lenth, 2016)) of current speed were determined at 0.27 ± 0.01 m s^−1^ of unidirectional flow in the exercise tank fitted with water pumps and at 0.13 ± 0.01 m s^−1^ of non‐directional flow in the control tank (linear mixed effects model: *χ*^2^ = 387.37; d.f. = 1; *p* < 0.001). The variation in flow within each tank, however, was greater than suggested by these mean values. The interquartile range of flow current speed (m s^−1^) across all positions and repeated measurements is given in File S1 Table S1. The circulation pumps in the exercise tank were activated 23.25–23.75 h daily, except for 15 min each day during which the animals in both treatments were fed to satiation with commercial trout feed, taking care that both tanks received exactly equal amounts and for an additional 20–30 min every second day for general maintenance and uneaten food and waste removal.

### 2.3 Sampling and measurements

*Salmo trutta* parr were sampled on five successive occasions. A first subsample of *n* = 6 parr were sampled before transfer into the individual round tanks, *i.e.* before treatment was initiated (hereafter referred to as group C00). Subsequent samples were taken from both, exercised (E) and control (C) cohorts after 4 weeks (hereafter referred to as groups E04, *n =* 6 and C04, *n =* 6, respectively), 10 weeks (E10, *n =* 6 and C10, *n =* 6), 20 weeks (E20, *n =* 6 and C20, *n =* 6) and 32 weeks (E32, *n =* 6 and C32, *n =* 6), creating subsamples of eight groups representing treatments (exercise and control) and age (weeks after treatment initiation) in addition to the initial control sample (C00). On each occasion, individual parr were dip‐netted from their holding tanks and immediately euthanized by pithing before being photographed and measured (described below). Body size prevented this method for parr after 32 weeks of treatment, so older parr were euthanized with an anaesthetic (MS‐222) overdose followed by pithing. After loss of equilibrium, parr were removed from the bath to be photographed for geometric morphometric analysis (described below) and then their spinal cord was severed using a surgical scalpel before further measurements were taken.

All husbandry and experimental procedures were approved by the University of Manchester's ethical committee and were covered by a UK Home Office project licence (licence number 40/3584, licence holder H.A.S.).

**Figure 1:**
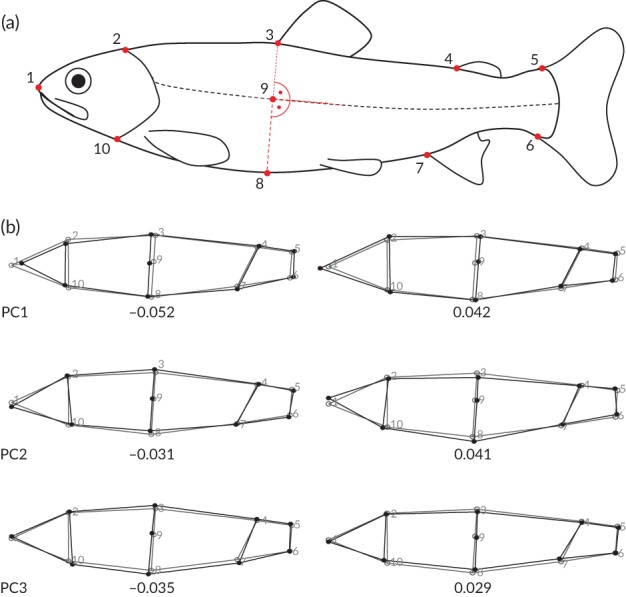
(a) Landmark positions (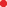) on Salmo trutta parr that were digitised twice and then averaged to minimize measurement error. (b) Shape changes associated with principal components (PCs) 1–3. PCs were derived from a between‐group PC analysis of Procrustes superimposed landmarks. 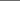, Consensus shape with numbered landmark positions; 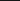, Shape changes associated with each PC. Shape changes are scaled to observed PC scores: Left hand side shape changes (back outlines) are scaled to the minimum value observed across the sample on each respective PC (shown below the image) and right hand side shape changes (black outlines) are scaled to the maximum value observed across the sample on each respective PC. PC1 describes a change in head size, PC2 describes dorso‐ventral arching of the body and PC3 describes changes in overall robustness and body depth

For geometric morphometric analyses, individual parr were photographed lying on their right side, facing left on a white tray, next to a measuring tape for scale. Ten landmarks outlining common shape features (Figure 1(a)) were digitised in these photographs using ImageJ 1.49v (http://imagej.nih.gov/ij). Landmarks were digitised twice and later averaged for each individual to minimize measurement error.

After photographing, additional measurements of standard length (*L*_S_, cm) and body mass (*M*, wet weight in g) were taken in order to monitor growth and physiological condition of exercised and control individuals throughout the experiment. Exponential regressions of *M* against *L*_S_ (weight–length relationship *M* = *a* $L_{S}^{b}$, WLR; Froese, 2006) were used to derive and compare specific growth parameters (slope *b* and intercept *a*) for the exercised and control cohorts (each including the *n =* 6 C00 individuals as a common origin). An exponent *b* > 3 is indicative of a fish becoming fatter (of increased girth) relative to increases in *L*_S_. The cohort‐specific growth parameters were used to compute the mean condition‐at‐length, $\bar{K}$ = 100a$L_{S}^{b–3}$ (Froese, 2006) for each individual under the respective treatment. The more commonly used Fulton's condition factor *K* = *M*(*L* ^3^)^−1^ (Froese, 2006) is a size‐corrected indicator of fish physiological condition and assumes the cube law. $\bar{K}$_,_ used here, serves as a size‐specific indicator for deviations in shape and physiological condition of subpopulations from the normal cubic mass–length relationship and is therefore most suitable for inter‐population comparisons (Froese, 2006). $\bar{K}$ was used here due to its ability to illustrate an elongation in shape *v.* an increase in girth or body depth with length (Froese, 2006). This allowed for a more direct comparison with the geometric morphometric analyses performed in this study than provided by most other condition indices.

Caudal‐fin area (*A*_CF_, cm^2^) was measured from photographs using ImageJ software and subsequently used to calculate the dimensionless *A*_CF_:*L*_S_^2^ (*L*_S_ was squared to account for extraneous scaling artefacts).

### 2.4 Statistical analyses

Statistical analyses were carried out using MorphoJ 1.06d (Klingenberg, 2011) and R 3.3.1 GUI 1.68 Mavericks build (http://www.r-project.org).

#### 2.4.1 *Geometric morphometric analyses*

Duplicate landmark coordinates were imported into the integrated geometric morphometric analysis software MorphoJ (Klingenberg, 2011). MorphoJ is a complete software package designed specifically for multivariate analyses of morphological landmarks on biological objects. In MorphoJ, duplicate landmarks were Procrustes superimposed, removing the effects of size and rotation to isolate shape features (Klingenberg, 2011). Procrustes superimposed duplicate landmark coordinates were averaged for each individual (full data set; *n =* 54 observations) and then for each group, reducing the data set to nine observations (reduced data set). A principal component analysis (PCA) was performed on the reduced data set. PCA creates linear combinations of variables, principal components (PC), reducing the dimensionality of the data and identifying the variables (*i.e.*, shape changes) associated with most variance in the data. The PC scores derived from this analysis were then used to perform a secondary PCA on the full dataset (averaged only for each individual). This two‐step PCA procedure improves the accuracy of measurements and therefore enhances the chance of finding subtle shape differences when samples originate from within the same species (*e.g.* different developmental stages) and the sample size is small (Klingenberg, 2014). Shape changes associated with the resulting secondary PCs were visualised and PC scores were exported for further analysis in R. PC2 was associated with dorso‐ventral arching (Figure 1(b)), an artefact of positioning the highly flexible fish bodies for photographs and not associated with biologically relevant shape features (Valentin *et al*., 2008). The data set of PC scores was corrected for this artefact using a procedure similar to Burnaby's (1966) size correction (Supporting Information S2 Statistical analyses), effectively removing the extraneous variable from the data set (Valentin *et al*., 2008). The remaining, corrected PC scores were subsequently analysed in a linear discriminant analysis in R (mass package (Venables & Ripley, 2002)) and group classifications were quantified with pairwise Mahalanobis distances (hdmd package 1.2 in R) and permutated Hotellings *T* ^2^‐ tests (Hotelling package 1.0–3 in R; Supporting Information S Statistical analyses).

#### 2.4.2 *Mass–length relationships and condition‐at‐length*

The growth parameters (slope *b* and intercept *a*) for the log mass–log length relationship of exercised *v.* control cohorts of *S. trutta* parr were compared using the general linear model car package 2.1–3 in R (Fox & Weisberg, 2011). Both cohorts included the six C00 individuals (pre‐treatment initiation controls) as a common origin. Condition‐at‐length $\bar{K}$ (computed from separately derived, cohort specific growth parameters) was compared between treatment–age groups using the car package 2.1–3 in R (Fox & Weisberg, 2011), with treatment (exercise, control) and age (weeks 4, 10, 20, 32 after treatment initiation) as categorical explanatory variables. Least‐squares means (R package lsmeans 2.23–5 (Lenth, 2016)) were computed as *post hoc*, pairwise comparisons between groups.

#### 2.4.3 *Caudal fin area measurements*

The caudal‐fin area–standard length ratio (*A*_CF_:*L*_S_^2^) was compared between *S. trutta* parr after 20 and 32 weeks of treatment (exercise, E–control, C). In the 32 weeks age group, two out of six control individuals had damaged caudal fins and these individuals were excluded from subsequent analyses. No deviations from normality were detected for *A*_CF_:*L*_S_^2^ and ratios were subsequently compared between treatments (exercise *v.* control) of *S. trutta* parr after 20 and 32 weeks treatment, respectively, using Welch's two sample *t*‐tests. In addition, we compared *A*_CF_:*L*_S_^2^ between age groups (20 and 32 weeks) irrespective of treatment, again using Welch's two sample *t*‐test.

## 3 RESULTS

### 3.1 Geometric morphometrics

**Figure 2:**
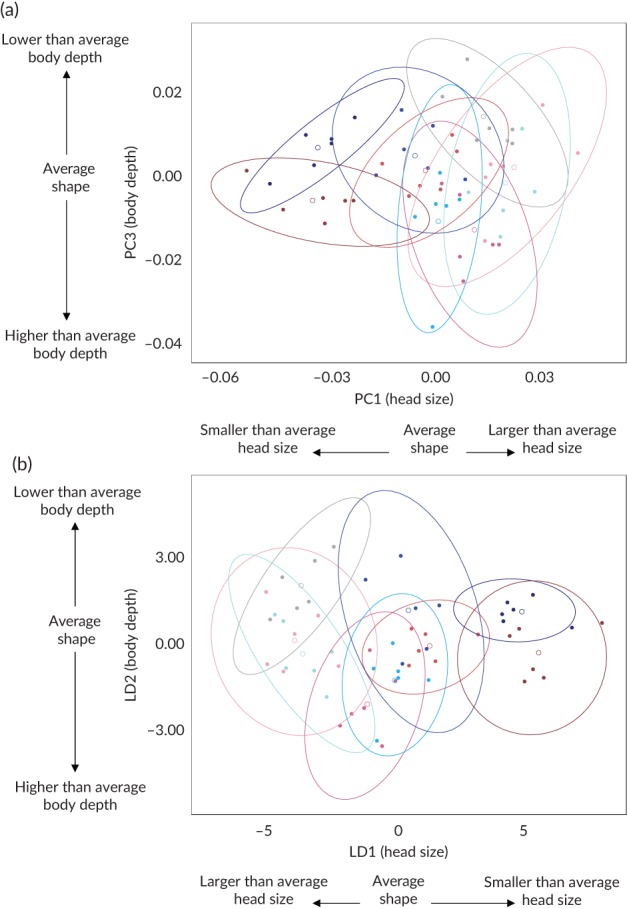
(a) Principal component (PC) (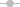) C00, (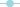) C04, (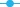) C10, (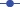) C20, (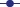) C32, (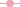) E04, (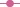) E10, (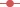) E20, and (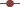) E32 and (b) linear discriminant (LD) scores for Salmo trutta treatment groups (C, control; E, exercise) across experimental weeks (i.e., age 00 (control sample before treatment initiation) to 32 (32 weeks of treatment); *n =* 6 individuals per group). PC1 and PC3, derived from a between‐group PC analysis of Procrustes superimposed landmarks corrected for the arching artefact (PC2). LD1 and LD2, derived from a LD analysis on the corrected principal component scores. Ellipses demarcate 95% confidence intervals; O, group centroids. *N.B.* The change of direction for head size on LD1 resulting from a negative association with PC1 (see Table 2)

The shape changes associated with the first three between‐group principal components (Figure 1(b)) cumulatively explained 76.9% of variance in the data (Table 1). PC1 described enlargement of head height at length, whereas PC3 was most strongly associated with reduced head and body depth. PC2 was associated with dorso‐ventral arching of the body and, as an artefact of positioning the flexible fish bodies for photographing, was removed mathematically before further analysis (see § 2.4.1). While individual loadings on PC1 described a progressive, age‐related shape change from a larger to smaller head size (Figure 2(a)), loadings on PC3 appeared to describe an increase in body depth of exercised as opposed to control groups in the later stages of the experiment (Figure 2(a)). This was partly confirmed by the results from linear discriminant analysis *(LDA)*: the coefficients from linear discriminants 1 and 2, cumulatively explaining 88.33% of variance in the data (Table 1) were most strongly associated with PCs 1 and 3, respectively (Table 2). LDA achieved 85.19% correct group classification and 62.96% cross‐validated correct classification for treatment groups across weeks (Figure 2(b) and Table 3). Between‐group Mahalanobis distances and permutated *T* ^2^ tests (Table S2 in File S1) confirmed a clear group separation along the age axis (weeks under treatment), associated with a decrease in head height/depth relative to lateral body depth (LD1, PC1; Figure 2(b)). Although body depth appeared to be greater in the exercised as opposed to control groups towards the end of the experiment (LD2, PC3; Figure 2(b)), the difference between same‐aged exercised and control groups was not statistically significant (*p* > 0.05; Table S2 in File S1).

**Table 1:** Variance explained by principal component analysis (PCA) on Procrustes superimposed landmarks on Salmo trutta parr and linear discriminant analysis (LDA) on the PC scores after correction for the arching artefact (PC2)

Between‐group PCA			Between‐group LDA		
PC	% Variance	Cumulative	LD	% Variance	Cumulative
1	40.53	40.53	1	78.74	78.74
2	24.3	64.83			
3	12.063	76.893	2	9.59	88.33
4	6.605	83.498	4	2.85	96.66
5	7.077	90.575	3	5.48	93.81
6	2.283	92.858	5	1.95	98.61
7	2.340	95.198	6	1.16	99.77
8	4.802	100	7	0.23	100
Total variance 0.00111746					

LDs and PCs in the same row share the highest correlation coefficients (see also Table 2).

**Table 2:** Coefficients derived from a linear discriminants (LD) analysis on PC scores from a between‐group PC analysis of Procrustes superimposed landmarks on Salmo trutta parr, corrected for the arching artefact (PC2)

PC	LD1	LD2	LD3	LD4	LD5	LD6	LD7
1	**−167.542**	−8.288	−3.359	−1.238	1.266	−1.084	0.331
3	−50.978	**135.098**	2.843	−7.214	5.396	−1.658	2.031
4	−35.763	16.565	40.427	**130.722**	3.53	−15.497	3.296
5	−57.077	−5.567	**171.722**	−33.709	16.095	20.402	−7.322
6	−16.868	10.79	64.857	−40.653	**−169.6**	−112.305	2.876
7	139.125	−58.136	41.611	−51.011	159.939	**−131.278**	14.126
8	−81.983	62.377	14.785	26.671	29.445	−53.529	**−122.749**

The largest coefficients, denoting the closest (negative or positive) association with the respective principal components (PC), are shown in bold.

**Table 3:** Cross‐validated linear discriminant (LD) classification results for Salmo trutta control (C) and exercise (E) treatment groups across experimental weeks 00 (control sample before treatment initiation) to week 32 (32 weeks of treatment) derived from a LD analysis on principal component (PC) scores from a between‐group PC analysis of Procrustes superimposed landmarks, corrected for the arching artefact (PC2)

	C00	E04	C04	E10	C10	E20	C20	E32	C32	Correct (%)
C00	**5**	1	0	0	0	0	0	0	0	83.33
E04	1	**2**	3	0	0	0	0	0	0	33.33
C04	1	1	**3**	1	0	0	0	0	0	50
E10	0	0	1	**4**	1	0	0	0	0	66.67
C10	0	0	0	2	**3**	1	0	0	0	50
E20	0	0	0	0	0	**5**	1	0	0	83.33
C20	0	0	0	0	1	1	**4**	0	0	66.67
E32	0	0	0	0	0	0	0	**3**	3	50
C32	0	0	0	0	0	0	0	1	**5**	83.33
Total cross‐validated										62.96
Total un‐validated										85.19

Numbers of correctly classified cases are shown in bold.

### 3.2 Mass–length relationships and condition at length

**Figure 3:**
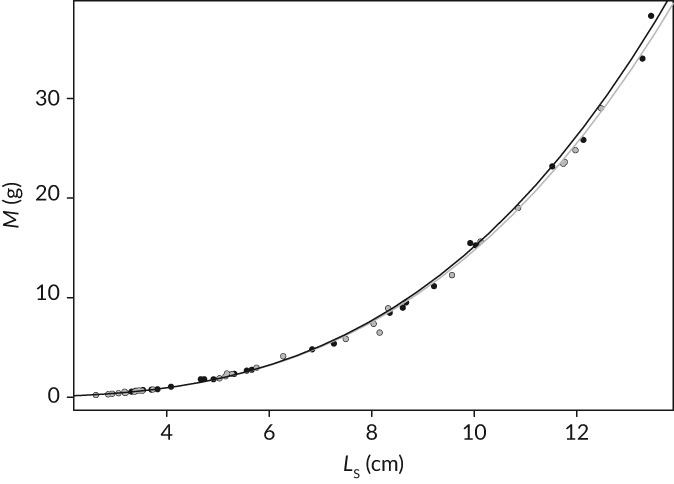
Mass–standard length (*M*–*L*_S_) relationships (MLR) determined for exercised (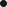) and control (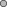) Salmo trutta cohorts over 0–32 weeks from treatment initiation. Each cohort included *L*_S_00 individuals (*n* = 6) as a common origin

**Figure 4:**
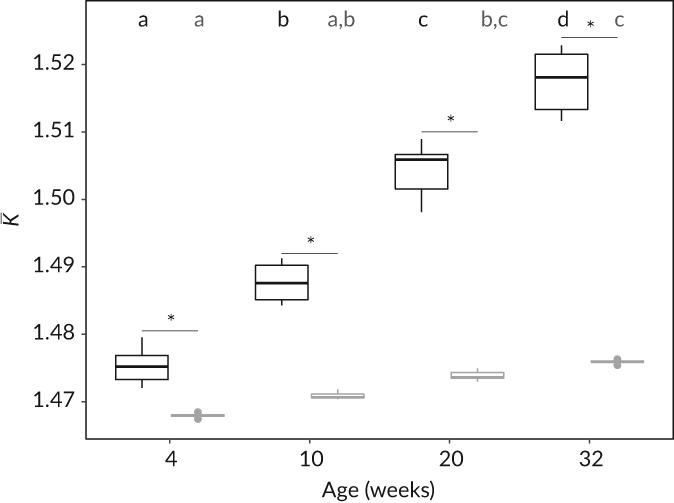
Box plots showing median (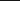), 25th–75th percentiles (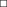) and range (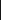) of Salmo trutta condition at length ($\bar{K})$ for exercised (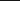) and control (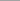) Salmo trutta cohorts across the experimental period (i.e.*,* age) weeks 4–32 after treatment initiation (*n* = 6 per group). *, significant differences of pairwise least‐squares means between exercised and control cohorts; different lower‐case letters (black, exercise; grey, control) denote significant differences of pairwise least‐squares means within treatments across the experimental period

A comparison of log mass‐log length relationships for the exercised *v.* control cohorts did not reveal a significant difference in the slopes (*F*_1,43_ *=* 0.21; *p* > 0.05) or intercepts dependent on treatment (*F*_1,43_ = 0.65; *p* > 0.05). Cohort‐specific regressions of *M* and *L*_S_ with the C00 individuals (*n =* 6) as a common origin described a mass–length relationship in the form of $M=0.0143L_{s}^{3.0242}$ for the exercised cohort and $M=0.0146L_{s}^{3.0044}$ for the control cohort (Figure 3). Therefore, exercised individuals were slightly (albeit not significantly) heavier for their length as they grew larger (stronger positive deviation of *b* from the cubic 3) than control individuals. Consequently, condition‐at‐length, $\bar{K}$, derived from the regression parameters for each cohort, differed across weeks (i.e.*,* age; *F*_3,39_ *=* 194.038, d.f. = 3, *p* < 0.001) as well as treatments (*F*_1,39_ *=* 864.99, d.f. = 1, *p* < 0.001) (Figure 4). In addition, the pattern of variation in $\bar{K}$ across weeks differed between treatments as indicated by the statistically significant interaction term (*F*_3,39_ = 85.803, d.f. = 3, *p* < 0.001). Specifically, $\bar{K}\;$was higher in exercised compared with control groups over the whole experimental period. Within treatments, $\bar{K}\;$consistently increased for exercised individuals over the experimental period, while for control individuals, $\bar{K}\;$only increased from week 4 to week 20, week 4 to week 32 and week 10 to week 32 (Figure 4).

### 3.3 Caudal‐fin size

**Figure 5:**
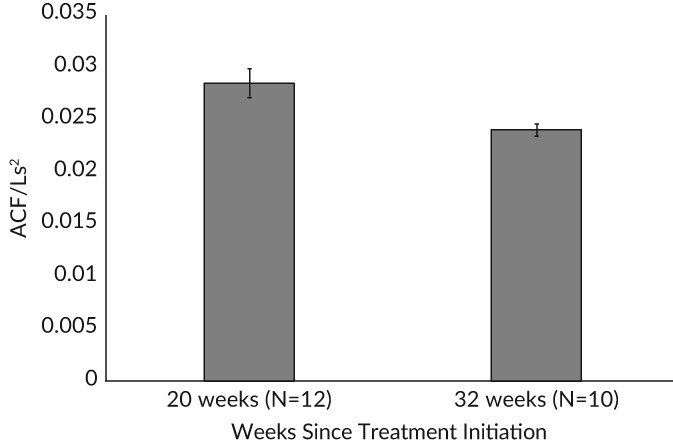
Caudal fin area: body length ratio (*A*_CF_:*L*_s_^2^; mean ± s.e.) measured from photographs of Salmo trutta parr at 20 and 32 weeks after exercise treatment initiation. *A*_CF_:*L*_s_^2^ values between the two groups were significantly different (Welch's two sample *t*‐test *p* < 0.05)

*A*_CF_:*L*_S_^2^ did not differ between treatments at 20 weeks (mean ± s.e.; exercised = 0.029 ± 0.002, control = 0.028 ± 0.002; *t* = 0.291, d.f. = 9.36, *p* > 0.05) or at 32 weeks (exercised = 0.024 ± 0.001, control = 0.024 ± 0.001; *t* = −0.147, d.f. = 8, *p* > 0.05) after treatment initiation. However, overall and irrespective of treatment, *A*_CF_:*L*_S_^2^ ratios were significantly higher after 32 weeks compared with 20 weeks (Figure 5) since treatment initiation (20 weeks = 0.028 ± 0.001, 32 weeks = 0.024 ± 0.001; *t* = 2.999, d.f. = 14.778, *p* < 0.01).

## 4 DISCUSSION

The geometric morphometric analysis performed in this study indicated that head height decreased (the head became more slender) with age, but no clear effect of flow condition on body shape in the lateral aspect was detected. However, consistent with one of our hypotheses, the flow treatment induced changes in robustness. A steeper mass–length trajectory of individuals reared under increased flow meant that $\bar{K}$ was continuously higher than that of the controls, indicating that exercised *S. trutta* parr were more robust at any given length. The individuals housed in the increased flow grew more robust (heavier, possibly rounder‐bodied) compared with control individuals over the course of the experiment, which may convey improved survivability through an increase in available energy reserves, or perhaps an increase in muscle mass for improved swimming ability. Although caudal‐fin area (relative to body length) increased as the parr developed, there was no detectable difference between treatments. Hence, a flow induced increase in the size of the caudal‐fin area was unsupported and there was no evidence of a flow induced shape change to the lateral aspect of the *S. trutta* parr.

Increased head size relative to body size was observed as fish grew from the typical larval to adult appearance. Because this change was observed irrespective of treatment, it was attributed to ontogenetic development. In addition to head size, PC analysis identified changes in body depth of the *S. trutta* parr. A streamlined body shape is thought to improve swimming ability and efficiency for feeding on patchily distributed prey in open water and during migration, while a deeper body shape improves burst swimming and manoeuvrability in structurally complex habitats with variable flow (Webb, 1984; Langerhans, 2008). The results showed some differentiation between exercised and control groups along the axis representing body depth (separation of centroids along LD2; Figure 2(b)), with exercised individuals showing increased body depth, but the effect was not statistically significant (Supporting Information Table S2). This study did not, therefore, provide evidence for an effect of exercise treatment on body shape features associated with manoeuvrability. However, the observed differences in $\bar{K}$, indicate that *S. trutta* parr subjected to the higher flow regime grew more robust (heavier, rounder) over time than parr reared in our control treatment. While geometric morphometric measurements in this study were limited to the lateral shape of the *S. trutta* parr, $\bar{K}$ describes changes in body depth and, or width with increasing length.

Previous studies investigating the effect of differential flow conditions on salmonid morphology have yielded clearer results compared with ours, but the direction of effects was often contradictory. For example, wild‐origin young‐of‐the‐year juvenile *S. trutta* reared in a hatchery under increased flow developed a more fusiform body shape compared with conspecifics reared in low flow, while exercised *S. salar* developed a deeper body shape (Pakkasmaa & Piironen, 2000). Pakkasmaa and Piironen (2000) attribute this discrepancy to species‐specific reaction norms. While this is probably the case, it is also possible that differences in resource availability confounded their results. Because food dispersed more rapidly in Pakkasmaa and Piironen's (2000) higher flow treatment, this cohort of fish was given an extra portion of food. It is not possible, therefore, to determine whether both treatments received equal amounts of resources. In the present study, this issue was avoided by interrupting flow during feeding while assuring that both exercise and control cohorts were fed the same amounts.

By further contrast to the Pakkasmaa and Piironen (2000) study, *S. salar* aged 2+ years were more robust in slower flow rather than faster currents, while body shape of younger (1+ years) individuals did not differ in response to different flow conditions (Páez *et al*., 2008). There may therefore be fundamental differences in the reaction norms of salmonids to environmental variation *(e.g.* water velocity) dependent on the age and developmental stage of the subjects (Grünbaum *et al*., 2008). Here, this question was addressed by comparing flow treatments over a long period (32 weeks).

It is possible that the sample size of *n =* 6 individuals in each treatment group of this study was too small to detect an effect of exercise on body depth using geometric morphometric methods and this possible limitation needs to be considered when interpreting the results. Alternatively, the variable flow conditions in the exercise treatment (Table S1) may have been insufficient to induce the rapid shape changes observed in previous studies where flow was strictly laminar (Fischer‐Rousseau *et al*., 2010; Grünbaum *et al*., 2007). However, in their natural, riverine habitats, salmonid parr are not subjected to laminar flow such as created in most laboratory experiments, but instead experience highly variable velocities, with flow shelters created by objects in the stream alongside rapid currents (Hinch *et al*., 2005), similar to the conditions in the exercise treatment of our experiment, where the flow was heterogeneous (Supporting Information Table S1). Variable flow is likely to be easier to implement in a hatchery setting compared with strictly laminar flow and this structural heterogeneity may be advantageous for growth, as shown here, and to maintain phenotypic diversity.

Comparisons between wild and captive salmonids have shown that the heterogeneous rearing conditions in the wild are responsible for significant behavioural, physiological and morphological differentiation. For example, wild, juvenile *S. trutta* had longer heads and an elongated postcranial trunk, making them more fusiform than hatchery‐reared individuals from the same genetic (wild‐origin) stock (Vehanen & Huusko, 2011). Release and recapture of hatchery‐reared juveniles showed that living in their natural, heterogeneous environment reversed some of the morphological effects induced in captivity (Vehanen & Huusko, 2011). Furthermore, in wild adult rainbow trout *Oncorhynchus mykiss* (Walbaum 1792) clear morphological differences were observed across several ecotypes and their response to induced flow as opposed to still water in experimental holding tanks differed (Keeley *et al*., 2007). Keeley *et al*. (2007) concluded that phenotypic plasticity is mainly caused by genetically controlled local adaptations (ecotypes), while environmental heterogeneity plays a secondary role in shaping inter‐population differences in morphology. The contrary results presented in many previous studies (Fleming *et al*., 1994; Poole *et al*., 2003; von Cramon‐Taubadel *et al*., 2005) may therefore be partly attributable to underlying genetic differentiation in the wild‐origin individuals and their distinctive response to the experimental treatments. This intra‐species diversity also illustrates the genetic heterogeneity in wild salmonids and the resultant high potential for rapid and flexible adaptation to local conditions (Garcia de Leaniz *et al*., 2007). In domesticated individuals, like the ones used in the present study, however, this adaptability is probably decreased.

In captivity, certain selective pressures, such as resource competition and predation, are absent. Together with possible effects of inbreeding depression (Jonsson & Jonsson, 2011), this can lead to rapid antagonistic selection and significant changes in the genetic makeup of captive fish (Araki *et al*., 2007; Araki *et al*., 2008). Indeed, it may be the high potential for phenotypic plasticity that allows wild salmonids to rapidly adapt to local environmental conditions (Garcia de Leaniz *et al*., 2007) and causes their rapid, yet ultimately detrimental adaptation to the conditions in captivity (Araki *et al*., 2007; Heath *et al*., 2003), reducing their genetic heterogeneity in the process (Blanchet *et al*., 2008). This raises the question whether captive‐reared and domesticated salmonids, such as the ones used in the present study, retain the same potential for phenotypic plasticity and adaptation to newly induced environmental fluctuations (*e.g.* altered flow conditions) that have been demonstrated for wild‐origin individuals (Garcia de Leaniz *et al*., 2007; Keeley *et al*., 2007). A lack of an effect on the lateral body shape and caudal fin of the parr perhaps suggests that phenotypic adaptability in the sample was indeed limited. However, the sample size used in this study was limited and future studies should aim to confirm this effect on a broader scale.

Although perhaps a tertiary consideration, this study suggests that despite the clear statistical advantages of utilising geometric morphometric analyses (Klingenberg, 2010), they require a relatively large sample size and are computationally complex and time consuming. Measurements of mass and length to compute condition factors such as $\bar{K}$ may therefore be more applicable to monitoring fish development in large‐scale industrial settings such as hatcheries.

In summary, this study is, to our knowledge, the first to investigate the long‐term response of domesticated, young‐of‐the‐year *S. trutta* parr to water flow under controlled conditions. The results show that increased, variable flow improves physiological condition and mass with increasing length, although morphological differentiation of lateral body shape and the caudal fin as the main propulsor during locomotion was limited between treatments. Crucially, these results indicate that the adaptability of domesticated salmonids to variable environmental conditions may be limited, highlighting the importance of habitat enrichment and structural heterogeneity throughout development in captivity for sustainable species management.

## ACKNOWLEDGEMENTS

We are indebted to K. Ozolina, K. Rose and N. Thavarajah for their assistance with data collection. We would like to thank the anonymous reviewers for their constructive comments on this manuscript.

## Supporting information

File S1. Supporting informations.FIGURE S1 Schematic depicting the measurement positions in the experimental holding tanks. Numbers 1–4 show the equidistant positions in the tank at which measurements were taken at three depths: A, surface; B, mid‐depth; C, tank base. 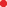, Positions behind the central tube element; 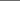, position of circulation pump in the exercise tank.
**TABLE S1** Interquartile range of flow speeds (m/s) taken eight times at 36 positions (Figure S1) in each tank covering three horizontal and three vertical depths at four equidistant positions along the circular water channel at the start of the experimental period.
**TABLE S2** Group separation through linear discriminants. Linear discriminant scores were used to compute between‐group squared Mahalanobis distances, *D* ^2^, and to perform pairwise Hotelling's *T* ^2^ tests with 10,000 permutations for Salmo trutta treatment groups (C, control; E, exercise) across experimental weeks (*i.e.* age 00 (control sample before treatment initiation) to 32 (32 weeks of treatment)). LDs were derived from an LDA on principal component scores from a between‐group PCA of Procrustes superimposed landmarks, corrected for the arching artefact (PC2).
