# Supplementary material for: Body shape and robustness response to water flow during development of brown trout Salmo trutta parr
Source: J Fish Biol. 2018 Sep 21;93(2):360–9. doi: 10.1111/jfb.13772 (PMC6174970; doi:10.1111/jfb.13772)
Supplement: Supplementary file 1 — File S1. Supporting informations. FIGURE S1 Schematic depicting the measurement positions in the experimental holding tanks. Numbers 1–4 show the equidistant positions in the tank at which measurements were taken at three depths: A, surface; B, mid‐depth; C, tank base. , Positions behind the central tube element; , position of circulation pump in the exercise tank. TABLE S1 Interquartile range of flow speeds (m/s) taken eight times at 36 positions (Figure S1) in each tank covering three horizontal and three vertical depths at four equidistant positions along the circular water channel at the start of the experimental period. TABLE S2 Group separation through linear discriminants. Linear discriminant scores were used to compute between‐group squared Mahalanobis distances, D 2, and to perform pairwise Hotelling's T 2 tests with 10,000 permutations for Salmo trutta treatment groups (C, control; E, exercise) across experimental weeks (i.e. age 00 (control sample before treatment initiation) to 32 (32 weeks of treatment)). LDs were derived from an LDA on principal component scores from a between‐group PCA of Procrustes superimposed landmarks, corrected for the arching artefact (PC2). [file JFB-93-360-s001.docx]

**SUPPORTING INFORMATION**

**S1 |** **FLOW SPEED MEASUREMENTS**

Water flow was determined to the nearest 0.01 m s^–1^ in both tanks at the start of the experiment using a dip-in flow probe (HFA, Höntzsch Instruments GmbH; www.hoentzsch.com) at 36 positions, covering three horizontal by three vertical depths at four equidistant positions along the circular water channel (Figure S1). A total of eight repeated measurements, spread over the initial 12 weeks of treatment, were used to determine the overall difference in current speed between the tanks. From a linear mixed effects model(R 3.3.1 GUI 1.68 Mavericks build (www.R-project.org), lme4 package 1.1-12 (Bates *et al.*, 2015) and car package 2.1-3 (Fox and Weisberg, 2011)), controlling for repeated measurements at each of the 36 positions, adjusted means (± S.E.; lsmeans package 2.23-5 (Lenth, 2016)) of current speed were determined at 0.27 ± 0.0087 m s^–1^ of unidirectional flow in the exercise tank fitted with water pumps and at 0.13 ± 0.0087 m s^–1^ of non-directional flow in the control tank (linear mixed effects model: $\chi^{2}$ = 387.37; d.f. = 1; *P* < 0.001). The interquartile range of flow current speed (m s^–1^) across all positions and repeated measurements is given in Table SI.

**S2 | STATISTICAL ANALYSES**

To correct geometric morphometric principal component (PC) scores for the extraneous effect of arching in the flexible fish bodies (PC2), PC scores were projected onto a complementary subspace defined using matrix algebra in the form of : $L= I_{q}- f_{1}\left( f_{1}^{'}f_{1} \right)^{-1}f_{1}^{'}$. For *q* variables, $f_{1}$ is a *q* x 1 column vector representing the variable that is to be removed from the data set (here, scores on PC2), $f_{1}^{'}$ is the transpose of $f_{1}$, $I_{q}$is a *q* x *q* identity matrix and *L* is a *q* x *q* matrix of rank *q*-1 (Rohlf and Bookstein, 1987). Multiplying *L* by the data matrix then generates a data set where variation is orthogonal to the vector $f_{1}$, effectively removing the extraneous variable from the data set (Valentin *et al.*, 2008).

To assess whether the shape changes associated with the corrected PCs could serve as classifiers for the different treatment–age groups, the remaining PC scores were analysed in a linear discriminant analysis (LDA) using the mass package in R (Venables and Ripley, 2002). Similar to PCA, LDA creates linear combinations of variables, linear discriminants (LD), which can then be used to assign each individual to its group of origin. If group separation by the linear discriminants (in this case, shape changes associated with PCs) is sufficient, assignment of observations into their groups of origin is successful, *i.e*. correct group classification is achieved. By combining PCA to reduce dimensionality and determine the main shape changes with LDA for classification, we could therefore assess whether shape changes are group specific, *i.e*. caused by treatment or age of the *Salmo trutta* parr. To quantify group separation along the linear discriminants (Table SII), pairwise Mahalanobis distances (*D*^2^) [R: hdmd package 1.2 (www.cran.r-project.org/package=HDMD)] were calculated and compared using pairwise between-group Hotelling’s *T*^2^ tests with 10 000 permutations (Hotelling package 1.0-3 in R(www.cran.r-project.org/package=Hotelling)]. Hotelling’s *T*^2^ statistic differs by a constant factor from the Mahalanobis distances *D*^2^ and therefore gives a reliable indication of their statistical significance (C. Klingenberg, pers. comm.).

**FIGURE S1**. Schematic depicting the measurement positions in the experimental holding tanks. Numbers 1–4 show the equidistant positions in the tank at which measurements were taken at three depths: A, surface; B, mid-depth; C, tank base. <- - –, Positions behind the central tube element; ⬇, position of circulation pump in the exercise tank.

**TABLE S1** Interquartile range of flow speeds (m s^–1^) taken eight times at 36 positions (Figure S1) in each tank covering three horizontal and three vertical depths at four equidistant positions along the circular water channel at the start of the experimental period

| **Tank** | **Minimum** | **25th Percentile** | **Median** | **75th Percentile** | **Maximum** | |
| --- | --- | --- | --- | --- | --- | --- |
| Exercise | 0.00 | 0.21 | 0.24 | 0.29 | | 0.74 |
| Control | 0.00 | 0.00 | 0.19 | 0.21 | | 0.26 |

**TABLE S2** Group separation through linear discriminants. Linear discriminant scores were used to compute between-group squared Mahalanobis distances, *D*^2^, and to perform pairwise Hotelling’s *T*^2^ tests with 10 000 permutations for *Salmo trutta* treatment groups (C, control; E, exercise) across experimental weeks (*i.e*. age 00 (control sample before treatment initiation) to 32 (32 weeks of treatment)). LDs were derived from an LDA on principal component scores from a between-group PCA of Procrustes superimposed landmarks, corrected for the arching artefact (PC2).

|  |  | **C00** | **E04** | **C04** | **E10** | **C10** | **E20** | **C20** | **E32** |
| --- | --- | --- | --- | --- | --- | --- | --- | --- | --- |
| E04 | *D*^2^  $T_{7,4}^{2}$  *P* | 5.43  28.64  > 0.05 |  |  |  |  |  |  |  |
| C04 | *D*^2^  $T_{7,4}^{2}$  *P* | 6.08  41.96  > 0.05 | 4.58  41.68  > 0.05 |  |  |  |  |  |  |
| E10 | *D*^2^  $T_{7,4}^{2}$  *P* | 8.35  161.57  < 0.05 | 4.37  _d.f. 7,5_ 78.29  > 0.05 | 4.82  _d.f. 7,5_ 113.77  < 0.05 |  |  |  |  |  |
| C10 | *D*^2^  $T_{7,4}^{2}$  *P* | 8.24  321.61  < 0.01 | 5.58  131.28  < 0.05 | 5.37  98.35  > 0.05 | 3.13  31.31  > 0.05 |  |  |  |  |
| E20 | *D*^2^  $T_{7,4}^{2}$  *P* | 8.38  343.78  < 0.01 | 6.2  152.83  < 0.05 | 4.69  755.09  < 0.01 | 5.5  159.67  < 0.05 | 5.57  113.42  < 0.05 |  |  |  |
| C20 | *D*^2^  $T_{7,4}^{2}$  *P* | 7.26  108.86  > 0.05 | 4.89  87.33  > 0.05 | 5.78  327.61  < 0.001 | 9.05  85.59  > 0.05 | 4.25  25.64  > 0.05 | 3.45  45.59  > 0.05 |  |  |
| E32 | *D*^2^  $T_{7,4}^{2}$  *P* | 10.12  827.69  < 0.01 | 9.48  272.07  < 0.05 | 8.71  437.9  < 0.01 | 5.45  170.03  < 0.05 | 5.03  204.99  < 0.05 | 4.26  216.49  < 0.01 | 6.02  113.48  > 0.05 |  |
| C32 | *D*^2^  $T_{7,4}^{2}$  *P* | 8.12  350.5  < 0.01 | 8.22  309.75  < 0.01 | 9.24  1273.28  < 0.001 | 7.97  190.4  < 0.05 | 7.19  156.8  < 0.05 | 5.87  340.7  < 0.01 | 5.78  384.61  < 0.05 | 1.93  26.07  > 0.05 |

REFERENCES

Bates, D., Maechler, M., Bolker, B. & Walker, S. (2015). Fitting linear mixed-effects models using lme4. *Journal of Statistical Software* **67**, 1-48. doi: arXiv:1406.5823.

Fox, J. & Weisberg, S. (2011). *An {R} companion to applied regression*. Thousand Oaks, CA: Sage.

Lenth, R. V. (2016). Least-squares means: R package lsmeans. *Journal of Statistical Software* **69**, 1-33. doi: 10.18637/jss.v069.i01.

Rohlf, F. J. & Bookstein, F. L. (1987). A comment on shearing as a method for size correction. *Systematic Zoology* **36**, 356-367. doi: 10.2307/2413400.

Valentin, A. E., Penin, X., Chanut, J. P., Sevigny, J. M. & Rohlfk, F. J. (2008). Arching effect on fish body shape in geometric morphometric studies. *Journal of Fish Biology* **73**, 623-638. doi: 10.1111/j.1095-8649.2008.01961.x.

Venables, W. N. & Ripley, B. D. (2002). *Modern applied statistics with S*. New York: Springer.
